# Measurement of Parity Violation in np Capture: the NPDGamma Experiment

**DOI:** 10.6028/jres.110.024

**Published:** 2005-06-01

**Authors:** Shelley A. Page, J. D. Bowman, R. D. Carlini, T. Case, T. E. Chupp, K. P. Coulter, M. Dabaghyan, D. Desai, S. J. Freedman, T. R. Gentile, M. T. Gericke, R. C. Gillis, G. L. Greene, F. W. Hersman, T. Ino, S. Ishimoto, G. L. Jones, B. Lauss, M. B. Leuschner, B. Losowski, R. Mahurin, Y. Masuda, G. S. Mitchell, H. Nann, S. I. Penttila, W. D. Ramsay, S. Santra, P.-N. Seo, E. I. Sharapov, T. B. Smith, W. M. Snow, W. S. Wilburn, V. Yuan, H. Zhu

**Affiliations:** University of Manitoba, Winnipeg, MB Canada R3T 2N2; Los Alamos National Laboratory, Los Alamos, NM 87545; Thomas Jefferson National Accelerator Facility, Newport News VA 23606; University of California, Berkeley, CA 94720-7300; University of Michigan, Ann Arbor, MI 48109-1120; University of New Hampshire, Durham, NH 03824; University of Tennessee, Knoxville, TN 37996-1200; University of California, Berkeley, CA 94720-7300; National Institute of Standards and Technology, Gaithersburg, MD 20899-0001; Los Alamos National Laboratory, Los Alamos, NM 87545; University of Manitoba, Winnipeg, MB Canada R3T 2N2; University of Tennessee, Knoxville, TN 37996-1200; University of New Hampshire, Durham, NH 03824; KEK National Laboratory, Tsukuba, Ibaraki 305-0801 Japan; Hamilton College, Clinton, NY 13323; University of California, Berkeley, CA 94720-7300; Indiana University Cyclotron Facility, Bloomington, IN 47408-1398; University of Tennessee, Knoxville, TN 37996-1200; KEK National Laboratory, Tsukuba, Ibaraki 305-0801 Japan; Los Alamos National Laboratory, Los Alamos, NM 87545; Indiana University Cyclotron Facility, Bloomington, IN 47408-1398; Los Alamos National Laboratory, Los Alamos, NM 87545; University of Manitoba, Winnipeg, MB Canada R3T 2N2; Indiana University Cyclotron Facility, Bloomington, IN 47408-1398; Los Alamos National Laboratory, Los Alamos, NM 87545; Joint Institute for Nuclear Research, Dubna, Russia; University of Dayton, Dayton OH 45469-1679; Indiana University Cyclotron Facility, Bloomington, IN 47408-1398; Los Alamos National Laboratory, Los Alamos, NM 87545; University of New Hampshire, Durham, NH 03824

**Keywords:** hadronic weak interaction, neutron capture, parity violation

## Abstract

The NPDGamma experiment will measure the parity-violating directional gamma ray asymmetry *A*_γ_ in the reaction $\vec{n}+p\to d+\gamma$. Ultimately, this will constitute the first measurement in the neutron-proton system that is sensitive enough to challenge modern theories of nuclear parity violation, providing a theoretically clean determination of the weak pion-nucleon coupling. A new beam-line at the Los Alamos Neutron Science Center (LANSCE) delivers pulsed cold neutrons to the apparatus, where they are polarized by transmission through a large volume polarized ^3^He spin filter and captured in a liquid para-hydrogen target. The 2.2 MeV gamma rays from the capture reaction are detected in an array of CsI(Tl) scintillators read out by vacuum photodiodes operated in current mode. We will complete commissioning of the apparatus and carry out a first measurement at LANSCE in 2004–05, which would provide a statistics-limited result for *A*_γ_ accurate to a standard uncertainty of ±5 × 10^−8^ level or better, improving on existing measurements in the neutron-proton system by a factor of 4. Plans to move the experiment to a reactor facility, where the greater flux would enable us to make a measurement with a standard uncertainty of ±1 × 10^−8^, are actively being pursued for the longer term.

## 1. Introduction

Despite the many remarkable successes of the Standard Model, hadronic weak interactions remain relatively poorly understood. On the theoretical side, calculations based on W and Z exchange between quarks are notoriously unreliable in the hadronic sector at low energies, where quarks are unavoidably bound by the strong interaction into nucleons and mesons. On the experimental side, studies of the weak nucleon-nucleon interaction are confined to measurements of parity-violating observables which constitute a unique but usually tiny signature of the weak interaction. This signature can be strongly enhanced by nuclear structure effects in heavier nuclei, but complications of the enhancement mechanisms have precluded a consistent determination of the most basic parameters of the weak nuclear potential despite a sizeable body of experimental data.

Theoretical descriptions of the weak nucleon-nucleon interaction are traditionally based on an effective meson exchange model in which the coupling constants for parity violating π, ρ, and ω meson exchanges set the scale for weak interaction effects and are uncertain to within factors of 2 to 3 on theoretical grounds [1]. Pion exchange mediates the only long range component of the interaction, and ought to be the dominant contribution to observed parity-violating effects in a wide range of nuclear systems. Early attempts to measure the weak pion nucleon coupling, $f_{\pi }^{1}$, were motivated by the unusually large sensitivity of the weak pion coupling to neutral currents, i.e., to the very existence of the Z boson as a carrier of the weak force, later demonstrated by the production of Z’s in high energy collisions at the European Center for Nuclear Research (CERN). The most precise limits on $f_{\pi }^{1}$ were obtained from measurements of the circular polarization of gamma rays from a well known parity-mixed doublet in ^18^F. Surprisingly small results were obtained in the ^18^F experiment, finding $f_{\pi }^{1}$ to be no larger than 10 % of the best theoretical estimate available, and consistent with zero [2]. On the other hand, a measurement of the anapole moment of ^133^Cs [3] indicates a surprisingly large value of $f_{\pi }^{1}$, which cannot be reconciled with the ^18^F data.

The neutron-proton system is the only two nucleon system that is sensitive to $f_{\pi }^{1}$ and can provide a clean measurement free of nuclear structure uncertainties. The up-down γ-ray asymmetry relative to the neutron spin direction in the reaction $\vec{n}+p\to d+\gamma$ at very low energy is sensitive almost exclusively to $f_{\pi }^{1}$, and this is the motivation for the NPDGamma experiment at LANSCE. Previous measurements [4] of *A*_γ_ failed to reach sufficient precision to test model predictions, and set only upper bounds that were less definitive than the ^18^F experiments noted earlier. Advances in techniques for producing high intensity beams of polarized, cold neutrons now make possible for the first time a measurement of *A*_γ_ and hence $f_{\pi }^{1}$ to within 10 % of model predictions. The asymmetry is predicted to be [5]

**Fig. 1:**
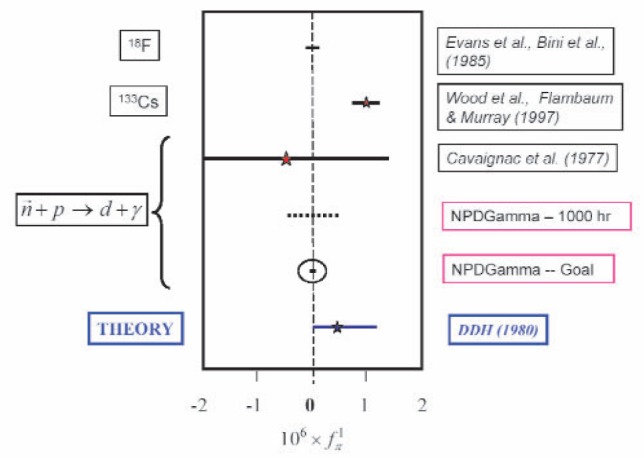
Experimental and theoretical limits on the weak pion-nucleon coupling $f_{\pi }^{1}$. The expected precision of an initial run of the NPDGamma experiment at LANSCE, assuming 1000 h of data, together with the statistics-limited final result planned for the experiment, are shown.

$A_{\gamma }=-0.11\;f_{\pi }^{1}=-5\;\times \;10^{-8}$ and we expect to be able to make a measurement to a standard uncertainty of ±1 × 10^−8^ or better, with systematic uncertainties at or below 5 × 10^−10^. The current experimental situation is illustrated in Fig. 1.

## 2. The NPDGamma Experiment

**Fig. 2:**
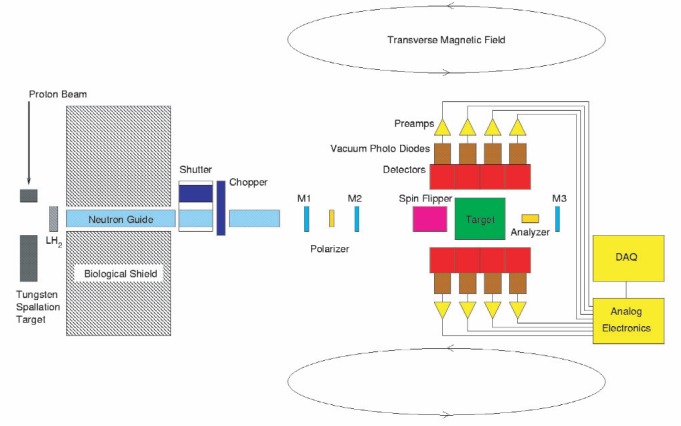
Schematic of the NPDGamma apparatus on flight path 12 at LANSCE (see text).

The NPDGamma experiment [6] is the first of a new program of fundamental electroweak symmetry experiments to be run at the Lujan Center spallation neutron source at LANSCE, which currently provides the highest intensity pulsed cold neutron source in the world for fundamental neutron physics. To measure the parity-violating gamma ray asymmetry in $\vec{n}+p\to d+\gamma$, we need an intense source of polarized neutrons in the meV energy range, a hydrogen target, a high-efficiency, large solid angle γ-ray detector, and a means of reversing the spin of the neutron beam without altering any other experimental conditions. At LANSCE, a 120 µA, 800 MeV proton beam pulsed at 20 Hz impinges on a tungsten spallation target; MeV neutrons emerging from the target are cooled in a liquid hydrogen moderator and transported via a supermirror guide to the experimental apparatus (Fig. 2), where they emerge from the (9.5 × 9.5) cm^2^ guide at 21 m from the source. The *m* = 3 supermirror guide [7] enhances the total neutron flux in the desired energy range 0 meV to 15 meV with respect to the Maxwellian distribution of neutrons emerging from the moderator. The pulsed nature of the beam enables the energies of the neutrons to be determined from their times of flight, which is an important advantage for diagnosing and reducing many types of systematic uncertainty.

**Fig. 3:**
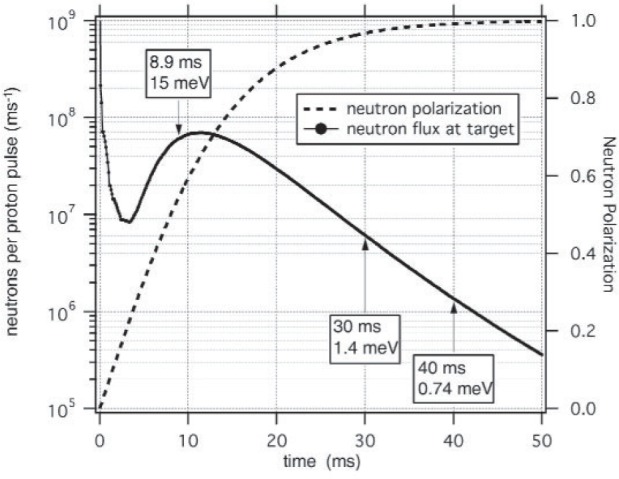
Monte Carlo simulation (NPDGamma proposal) of the neutron flux and polarization distributions versus time of flight, assuming a 10 cm diameter, 500 cm^3 3^He polarizing cell at 5 bar cm and 50 % polarization, achieved by spin exchange optical pumping of ^3^He.

Neutrons are polarized in the vertical direction by selective transmission through a polarized ^3^He gas cell which acts as a spin filter, producing an energy dependent polarization spectrum. Figure 3 shows a Monte Carlo simulation of the anticipated neutron beam intensity and polarization distributions at a target location 15 m from the source, for a 200 µA proton beam and a 5 bar cm ^3^He spin filter cell polarized at 65 %. (These conditions, from the experimental proposal in 1999, are somewhat more optimistic than the present running conditions, as discussed in Sec. 3.) The neutron beam intensity is measured with a ^3^He ionization chamber upstream and downstream of the polarizer cell, and again at the end of the beamline with a third ion chamber. The transmission of the ^3^He cell serves as an online measurement of its polarization and hence that of the neutron beam. A uniform vertical guide field, *B*_o_ = 1 mT preserves the neutron beam polarization as it is transported to the liquid hydrogen target, where the incident neutrons are captured to produce the 2.2 MeV gamma rays of interest.

Low energy neutrons depolarize rapidly in orthohydrogen, while those below 15 meV retain their polarization in a parahydrogen target; hence, it is important to ensure that the liquid hydrogen target is prepared and maintained with the very low equilibrium ortho-hydrogen concentration of 0.1 %. On-line monitoring of the target transmission provides a check on the target conditions, since the scattering cross-sections for ortho and para hydrogen differ by about a factor of 20 in the energy range of interest. Approximately 60 % of the beam neutrons will be captured in the target; those that scatter through the target walls will be absorbed in a ^6^Li liner surrounding the target vessel, and the remaining 15 % will be transmitted to the ionization chamber at the end of the beamline for diagnostic purposes.

The 2.2 MeV γ rays from neutron capture in the target are detected with an array of 48, (15 cm × 15 cm × 15 cm) CsI(Tl) crystals [8] surrounding the target. The detectors are read out in current mode by vacuum photodiodes coupled via low noise current-to-voltage preamplifiers to transient digitizers sampling the diode signals every 10 µs. The time of flight information from the CsI detectors allows the γ-ray asymmetry *A*_γ_ with respect to the neutron spin direction to be deduced as a function of incident neutron energy from the angular distribution: $\frac{d\omega }{d\Omega }=\frac{1}{4ð}\;\left(1+A_{\gamma }\cos\theta \right)$, where *θ* is the angle between the neutron spin and the direction of emission of the gamma ray. *A*_γ_ should be constant for all of the cold neutrons in the beam, but the experimental asymmetry will reflect the energy dependence of the beam polarization as illustrated in Fig. 3. During NPDGamma data taking, a beam chopper upstream of the apparatus eliminates frame overlap by blocking very slow neutrons from the tail of the preceding beam pulse, and also cuts off each beam pulse after 33 ms in order to permit a beam-off background measurement in the γ detectors to be made as part of the normal data taking cycle.

**Fig. 4:**
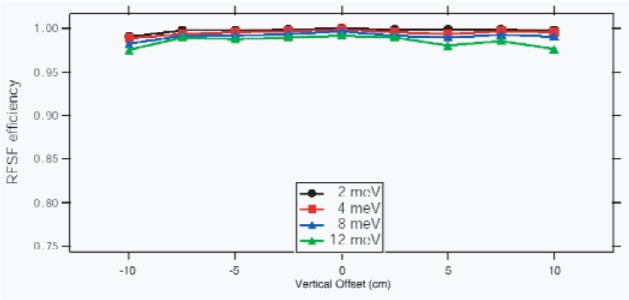
RF spin flipper efficiency, measured in a test run on FP11A at LANSCE.

A resonant radio frequency (RF) spin flipper, consisting of a 30 cm diameter by 30 cm long solenoid whose magnetic field amplitude is tailored as a function of time of flight to flip neutron spins of all energies, is located upstream of the target. With the use of a spin flipper, the up-down γ-ray asymmetry can be determined for each detector, effectively imposing full up-down geometrical symmetry on the apparatus. The spin flipper reverses the direction of the neutron spin on successive beam pulses according to an 8-step [+ − − + − + + −] reversal pattern, which cancels systematic drifts of detector efficiencies and electronic gains to 2nd order. Note that the asymmetry measurements are insensitive to noise at 60 Hz and harmonics, because the beam pulses are synchronized to 60 Hz. The spin flipper current supply is switched to a dummy load on alternate beam pulses when the flipper is “off” to keep the experimental conditions as constant as possible under the two spin flipper operational states. The spin flipper efficiency has been measured in tests runs to be in excess of 98 % across a large area as appropriate to the beam size in our experiment, as illustrated in Fig. 4.

The statistical uncertainty in the measurement of *A*_γ_ is ultimately determined by counting statistics, set by the beam intensity, the detector solid angle, and the counting time. The gamma ray detectors and low noise preamplifiers have been designed to ensure that sources of instrumental noise are small compared to this limit. The preamplifier noise alone is 100 times smaller than counting statistics at the rates that will be encountered during the *A*_γ_ measurements.

An exhaustive Monte Carlo study of possible systematic uncertainties has been carried out in the course of preparing this experiment. Care has been taken to identify all possible sources of uncertainty, to minimize the sensitivity of the apparatus, and to work out a program of ancillary measurements to quantify individual uncertainty sources. The overall conclusion of these studies is that it should be possible to measure *A*_γ_ to a standard uncertainty of ±0.5 × 10^−8^ with systematic uncertainties no larger than 10 % of the statistical uncertainty quoted above. Several key features of the experimental design should be noted here, namely:

1. Three independent magnetic field reversals can be employed to manipulate the neutron spin and should give identical results for *A*_γ_ (^3^He cell, RF spin flip, holding field in the experimental area);
2. the pulsed beam allows systematic effects to be isolated by their different time of flight dependences;
3. the use of vacuum photodiodes for detector readout reduces the gain sensitivity to magnetic fields as compared to conventional photomultiplier tubes by four orders of magnitude, and the detector gains are essentially independent of bias voltage;
4. the depolarization of the beam above 15 meV allows a number of systematics associated with interaction of polarized neutrons in the target to be isolated;
5. the very small value of the electronic noise compared to counting statistics (1/100) makes it possible to test for instrumental effects with a standard uncertainty of 10^−9^ on a timescale of 1 day.

Systematic uncertainties arising from interactions of the neutron spin are potentially the most serious for the experiment, including a number of reactions that take place in the hydrogen target in parallel with the $\vec{n}+p\to d+\gamma$ reaction. Spin dependent effects can lead to either up-down or left-right asymmetries which could leak into the up-down signal from which *A*_γ_ is deduced. To limit contributions from left-right asymmetries at or below 5 × 10^−10^, we require a means of determining the detector alignment with respect to the neutron spin direction to 20 mr or better. The alignment will be verified by scanning the detector array transverse to the beam by a few millimeters horizontally and vertically with the target in place and measuring the effective γ yield in each detector as a function of the array position.

The parity-conserving transverse analyzing power *A*_y_ in np elastic scattering gives rise to a left-right centroid shift of the beam—this is mitigated by the very low energy of the beam with an estimated analyzing power *A*_y_ ≈ 2 × 10^−8^, and the symmetry and alignment tolerances of the detector array yield an estimated false up-down asymmetry of 2 × 10^−10^ from this effect. Asymmetries associated with the $\vec{n}+p\to d+\gamma$ reaction itself include effects of a small circular polarization of the gamma rays, and a left-right asymmetry arising from the correlation ***s***_n_·(***k***_γ_ × ***k***_n_), where ***s***_n_ is the neutron spin direction, ***k***_γ_ and ***k***_n_ are the gamma ray and neutron propagation directions respectively—both have been examined and will lead to false asymmetries at the 10^−10^ level or smaller in the *A*_γ_ measurement. A parity violating neutron spin rotation will be experienced by the beam as it propagates through the liquid hydrogen; estimates of the size of this effect amount to 6 µrad across the 30 cm target, but the scale of this effect is negligible compared to our alignment requirement of ±20 mrad between the neutron spin direction and the up-down symmetry axis of the gamma ray detectors.

A false asymmetry associated with the correlation ***s***_n_·***k***_β_ in neutron beta decay, where ***k***_β_ is the propagation direction of the beta particle, is reduced by the fraction of neutrons that decay in the target (10^−7^) and the fractional gamma yield for a typical electron from the decay, yielding an estimate for the false asymmetry below 10^−12^. A small contamination of deuterium in the target can produce gamma rays via $\vec{n}+d\to t+\gamma$ with a parity violating asymmetry estimated from theoretical calculations of the weak meson-nucleon coupling constants; this is a small asymmetry to begin with and is further reduced by the relative cross sections and target abundances to an estimated false asymmetry of 10^−10^. The reaction $\vec{n}{+}^{6}\mathrm{Li}\to^{7}\mathrm{Li}*\to \alpha +t$ will take place in the Li-loaded plastic neutron-absorbing target liner, which is needed to prevent neutron damage to the gamma detectors; a parity violating ***s***_n_·***k***_α_ correlation, where ***k***_α_ is the propagation direction of the alpha particle, followed by (*α*, *n*) reactions, can lead to false asymmetries in the CsI detectors, estimated at the 2 × 10^−11^ level. Electromagnetic Mott-Schwinger scattering of polarized neutrons in the hydrogen target can lead to a left-right asymmetry of order 10^−8^ which will lead to a false up-down asymmetry of order 10^−10^ under experimental conditions.

Effects associated with the polarization of the beam but not arising from the hydrogen target can be isolated by comparing target full and target empty runs. One such effect is a potential Stern-Gerlach steering of the neutrons in the vertical direction, arising from inhomogeneities in the vertical 10 G guide field. Estimates of this effect for a 10^−5^ mT (0.1 G) field change over the dimensions of the apparatus predict a false asymmetry of 10^−10^, which reverses when the direction of the guide field is reversed. Another class of effects leads to gamma ray asymmetries from beta decays of polarized nuclei produced by interactions of the beam with materials upstream of the hydrogen target. Numerical estimates of false asymmetries from neutron capture on ^27^Al, ^26^Mg, ^7^Li, ^19^F, ^18^O, and ^208^Pb have been made; the largest effect is a false asymmetry from ^27^Al at 6 × 10^−10^ with all others an order of magnitude or more smaller than this.

## 3. Progress Towards Data Taking

In previous test runs carried out using prototype electronics and data acquisition systems on flight path 11 A, we demonstrated that the noise in the asymmetry signal measured with LEDs illuminating the photodiodes was at least 1/10 the noise in the γ-ray asymmetries produced by polarized neutron capture in a CCl_4_ target. We also measured the cold neutron flux, which compared well to a Monte Carlo simulation carried out by LANSCE accelerator staff for the present source conditions. Fluctuations in the cold neutron flux relative to the intensity of the primary proton beam were also measured and found to be at least a factor 1/10 the maximum tolerable consistent with the statistical goal of the experiment. We also measured a known parity violating asymmetry following neutron capture on ^35^Cl [9] to be: *A*_γ_ = (−29.1 ± 6.7) × 10^−6^. This test run result, taken with a 1/10 scale apparatus and lower neutron flux than that of flight path 12, which we will use for the NPDGamma measurements, was consistent with a previously published value but acquired in only a few hours of running time. During NPDGamma data taking, we will periodically measure this asymmetry with a Cl target to monitor the consistency of the detector performance.

**Fig. 5:**
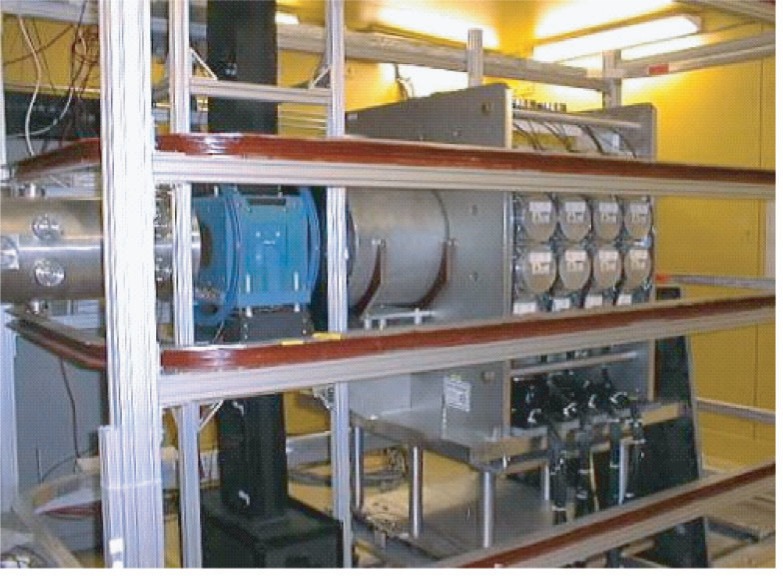
Photograph of NPDGamma apparatus installed in the new FP12 experimental cave, 2004. From left to right are shown the end of the neutron guide, the ^3^He polarizer assembly, the RF spin flipper, and the CsI detector array. The horizontal racetrack coils provide a uniform vertical guide field for the neutron spins. Present but not visible in this picture are two ^3^He ionization chambers for precision monitoring of the beam current and ^3^He polarizer characteristics, mounted on the downstream end of the neutron guide and immediately downstream of the ^3^He polarizer call, respectively. The liquid hydrogen target, whose vessel is completely surrounded by the gamma detector array, will be installed in the summer of 2004.

The new FP12 beamline for NPDGamma was completed late in 2003 [7], and the experimental apparatus, exclusive of the liquid hydrogen target, was installed in the new FP12 cave early in 2004 (Fig. 5). We have just completed a very successful first commissioning run, during which the beamline, chopper, beam monitors, magnetic guide field, ^3^He polarizer and analyzer cells, RF spin flipper, full CsI detector array and data acquisition systems were exercised. Following a tune up and commissioning of each element of the apparatus, time was devoted to measurements of parity violating asymmetries from solid targets that will contribute to backgrounds in the NPDGamma measurements, e.g., from the aluminum target vessel, and measurements with a boron target were used to confirm that the full array operates at the counting statistics limit [8]. Highlights of the commissioning run are discussed briefly below.

**Fig. 6:**
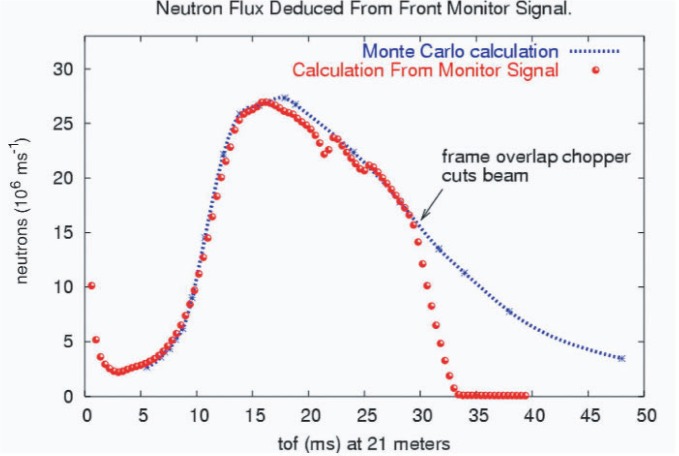
Neutron beam time of flight spectrum measured with a ^3^He ionization monitor mounted on the end of the flight path 12 neutron guide. The beam is intentionally blocked for *t* > 30 ms by a frame overlap chopper in order to provide an interleaved background measurement using the CsI detectors. The Monte Carlo simulation shown for comparison does not include distortions associated with aluminum Bragg edges due to windows in the beam.

Figure 6 shows the measured neutron time of flight distribution using the ^3^He ionization chamber mounted at the end of the flight path 12 neutron guide. Agreement with the Monte Carlo calculation as normalized to the measured moderator brightness [7] is excellent. Note that the measured peak flux is significantly lower than the goal simulation shown in Fig. 3—this is attributable to a lower moderator brightness as well as a lower production beam current in reality, as compared to initial forecasts when the LANSCE upgrade and the NPDGamma experiment were first proposed. Ultimately, the FP12 neutron flux will limit the precision in *A*_γ_ that we can obtain in a reasonable amount of running time at LANSCE.

**Fig. 7:**
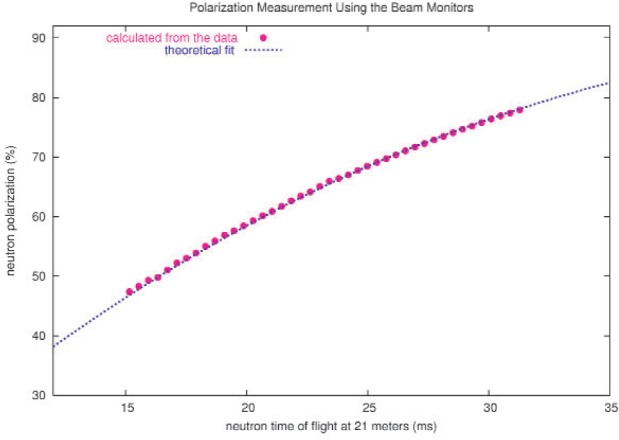
Neutron beam polarization deduced from ^3^He polarizer cell transmission measurements with the NPDGamma beam monitors during the 2004 commissioning run: $P_{n}=\sqrt{1-(T_{o}/T{)}^{2}}$ where *T*_o_ and *T* are the unpolarized and polarized transmissions and *P*_n_ is the neutron beam polarization. (The ^3^He polarization was not yet optimized when these data were taken.)

The experimental counting rate asymmetry is given by *ε* = *P*_n_*A*_γ_ where *P*_n_ is the beam polarization; the performance of the ^3^He spin filter cell therefore has a crucial influence on the statistical precision of the *A*_γ_ result. The large volume ^3^He cells for NPDGamma have been fabricated at NIST [10] and are approximately 10 cm diameter and 5 bar cm thick. The cells are polarized by spin exchange with polarized Rb vapor which is directly optically pumped using a diode laser array. ^3^He polarizations in excess of 60 % have been demonstrated in bench tests of these large cells. It should be noted that the spin filter technique for a thick polarizer cell can lead to neutron beam polarizations that are much higher than the ^3^He polarization and approaching 100 % for the slowest neutrons in the beam, as illustrated in Fig. 2. With precision beam monitors located upstream and downstream of the ^3^He polarizer cell, the neutron beam polarization can be inferred directly from a comparison of the polarized and unpolarized cell transmissions—this allows for a continuous on line measurement of the neutron beam polarization during NPDGamma data taking. An example under commissioning run conditions (the ^3^He polarization was not yet fully optimized) is shown in Fig. 7.

**Fig. 8:**
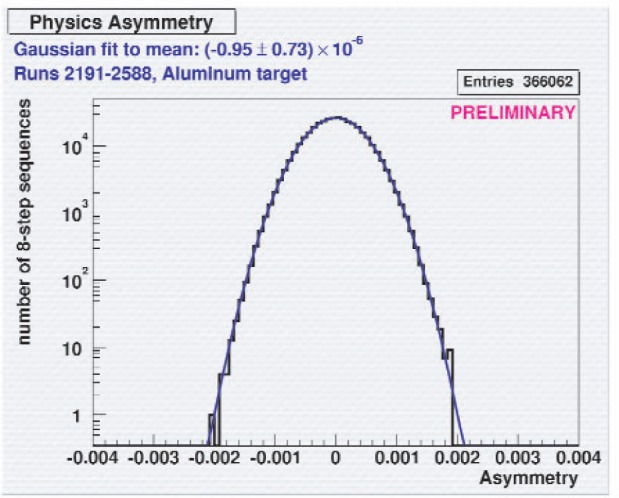
Preliminary measurement of the up-down parity violating γ asymmetry following neutron capture on aluminum. The histogram shows the asymmetry calculated from eight successive beam pulses with a spin reversal sequence arranged to minimize the sensitivity to first and second order gain drifts. Note the ideal Gaussian distribution of the data, over four orders of magnitude, with only very loose cuts placed on the neutron beam intensity. The measured asymmetry is small enough to proceed to hydrogen target data taking for NPDGamma without making a significant correction for this background effect.

During the commissioning run, we repeated our earlier measurements of the parity violating up-down γ asymmetry following neutron capture on ^35^Cl and confirmed the earlier results to much higher precision in a few hours of integration time. The NPDGamma detector array is such a precise instrument that we were able to use this small parity-violating asymmetry to tune the RF spin flipper, although use of a polarized ^3^He analyzer cell and a third beam monitor downstream of the cell is the preferred method. A preliminary result from the background studies using a solid aluminum target is shown in Fig. 8, which illustrates the superb performance of the CsI detector array. The γ-ray asymmetry histogram is seen to be Gaussian over at least four orders of magnitude, with only very loose cuts on the incident neutron beam intensity. The conclusion from this preliminary analysis is that the parity violating asymmetry following neutron capture on aluminum is small enough that it will result in a negligible background correction with the liquid hydrogen target running.

## 4. Summary and Future Outlook

The NPDGamma experiment has just completed a very successful first commissioning run on flight path 12 at LANSCE. We have demonstrated that the full CsI detector array, instrumented for current mode readout, achieves a statistical uncertainty consistent with counting statistics. This summer, the liquid hydrogen target will be installed, followed by first data taking as early as possible during the 2005 run cycle. We have determined that the physics asymmetry *A*_γ_ can be measured at LANSCE with the full NPDGamma apparatus with a statistical uncertainty of ±4 × 10^−4^ per beam pulse with 120 µA proton beam on the spallation target [9]—unfortunately this falls short of our original ±1 × 10^−4^ per beam pulse goal that would enable us to measure *A*_γ_ to 10 % of its predicted value in one calendar year of running time. Our test measurements [7] have shown that expectations of the available neutron flux from the upgraded LANSCE facility were too optimistic by almost a factor of four, with roughly equal contributions from reduced moderator brightness and reduced production beam current.

In view of the ultimate limit on the statistical accuracy that we can hope to achieve at LANSCE, the NPDGamma collaboration plans to complete commissioning of the apparatus and carry out a first measurement in 2005–2006, which would provide a statistics-limited result for *A*_γ_ accurate to a standard uncertainty of ±5 × 10^−8^ or better, improving on existing measurements in the neutron-proton system by a factor of four, as shown in Fig. 1. In the longer term, our aim is to move the experiment to the Fundamental Neutron Physics Beam Line at the Spallation Neutron Source, which would enable us to make a measurement with a standard uncertainty of 10^−8^.
